# Association Between Cardiovascular Disease and Cataracts: Protocol for a Systematic Review and Meta-Analysis

**DOI:** 10.2196/96552

**Published:** 2026-11-03

**Authors:** Jun Fai Yap, Pei Pei Heng, Wan Nur Syamimi Wan Mohamad Darani, Halizah Mat Rifin, Nor Asiah Muhamad, Mei Yin Pong, Muhammad Fadhli Mohd Yusoff

**Affiliations:** 1 Institute for Public Health, National Institutes of Health, Shah Alam, Malaysia, 60 33628764; 2 Molecular Pathology Unit, Cancer Research Centre, Institute for Medical Research, National Institutes of Health, Block C, Jalan Setia Murni U13/52, Seksyen U13 Setia Alam, Shah Alam, 40170, Malaysia; 3 Institute for Health Management, National Institutes of Health, Shah Alam, Malaysia; 4 Department of Social and Preventive Medicine, Faculty of Medicine, Universiti Malaya, Kuala Lumpur, Malaysia; 5 Sector for Evidence-Based Healthcare, National Institutes of Health, Shah Alam, Malaysia; 6 Department of Rehabilitation Medicine, Penang General Hospital, George Town, Penang, Malaysia; 7 Institute for Health Systems Research, National Institutes of Health, Shah Alam, Malaysia

**Keywords:** congestive cardiac failure, deep vein thrombosis, lens opacification, myocardial infarction, stroke

## Abstract

**Background:**

There is emerging evidence suggesting shared pathophysiological mechanisms between cataracts and cardiovascular disease (CVD), indicating a potential link between these 2 prevalent noncommunicable diseases. Oxidative stress, resulting from an imbalance between reactive oxygen species production and the body’s antioxidant defenses, contributes to lens protein aggregation in cataracts and lipid oxidation in atherosclerosis, a key feature of several CVD subtypes. This shared pathophysiology implies that systemic vascular health may influence cataractogenesis. However, the epidemiological evidence supporting this relationship remains inconclusive.

**Objective:**

The main objective of this systematic review is to synthesize existing evidence on the odds or risk of developing cataracts in adults with underlying CVD.

**Methods:**

This systematic review will adhere to the PRISMA-P (Preferred Reporting Items for Systematic Reviews and Meta-Analyses Protocols). Using the population, exposure, comparator, and outcome framework, this review will identify the association between established CVD and the risk of cataract development or needing cataract surgery. Systematic literature searches will be conducted in PubMed, Scopus, Web of Science, and Embase using MeSH terms from database inception to December 31, 2024. Original research articles involving adults (aged ≥18 years) that assess the statistical relationship between CVD and cataracts will be included. Two independent reviewers will screen studies, extract data, and appraise the studies’ quality using the Newcastle-Ottawa Scale. If deemed feasible, meta-analyses will be conducted using random-effects models to calculate pooled effect estimates, with heterogeneity assessed using the *I*^2^ statistic.

**Results:**

This study did not receive any external funding. The literature search, study screening, and data extraction will be conducted in 2026, and data analysis will be performed accordingly. The findings are anticipated to be published in 2027.

**Conclusions:**

This systematic review will provide a consolidated and up-to-date evaluation of the relationship between CVD and cataracts. The findings will highlight the potential importance of integrating cardiovascular risk assessment into routine ophthalmologic care. Continued refinement of study methodologies and standardization of outcome measures may be needed to address observed heterogeneity and strengthen future evidence, ultimately supporting more holistic management of patients with coexisting cardiovascular and ocular conditions.

## Introduction

Cataracts, characterized by progressive opacification of the eye’s crystalline lens, account for the largest proportion of preventable vision loss. In 2019, the age-standardized pooled prevalence estimate of cataracts was approximately 17.2%, reflecting the immense burden of this common eye disorder on public health [1]. With an aging population, the prevalence of cataracts is expected to rise substantially in the coming decades, placing increasing demands on health care systems worldwide [2]. Currently, no pharmacological treatments exist to slow or reverse lens opacity. The definitive treatment for cataracts remains surgical intervention, involving the extraction of the opacified lens and replacement with a synthetic intraocular lens. Beyond restoring visual acuity, cataract surgery enhances patients’ quality of life by improving cognitive function, mental health, and independence in daily activities [3].

Similarly, cardiovascular disease (CVD) has a profound societal impact, creating a substantial burden on the workforce worldwide [4,5]. The global prevalence rate of CVD was approximately 0.07% in 2019 [6], and it encompasses a wide range of prevalent medical disorders affecting the heart and blood vessels. For instance, acute coronary syndrome is characterized by reduced blood flow to the heart due to coronary artery blockages. Stroke, another major manifestation of CVD, occurs when blood supply to the brain is disrupted by either ischemia or hemorrhage. Collectively known as venous thromboembolism, deep vein thrombosis and pulmonary embolism are considered the third most common CVD following acute coronary syndrome and stroke. Deep vein thrombosis involves the formation of blood clots in deep veins, typically in the legs, which can travel via the bloodstream to the lungs, leading to a potentially fatal pulmonary embolism. Congestive cardiac failure, commonly referred to as heart failure, describes the heart’s inability to pump sufficient blood to meet the body’s metabolic demands due to structural or functional impairments in ventricular filling. Peripheral arterial disease, another underdiagnosed CVD subtype, results from narrowed or blocked arteries that restrict blood flow to the extremities, causing symptoms such as claudication pain, ulcers, or even gangrene. Together, these conditions highlight the extensive health burden posed by CVD and emphasize the need for comprehensive management strategies.

There is emerging evidence suggesting potential links between CVD and cataracts, highlighting shared pathophysiological mechanisms that may accelerate the onset of these noncommunicable diseases. For example, oxidative stress, a key driver of cataractogenesis, arises from an imbalance between reactive oxygen species production and antioxidant defenses, leading to the aggregation of damaged lens proteins [7]. This oxidative damage parallels processes in atherosclerosis in most CVDs, where reactive oxygen species–driven low-density lipoprotein oxidation induces plaque formation in blood vessels or endothelial dysfunction [8]. Such similarities highlight the potential of considering cardiovascular risk assessment in patients with newly diagnosed cataracts, promoting a holistic approach that addresses both ocular and systemic diseases.

Shared risk factors for CVD and cataracts, such as diabetes mellitus [9] or hypertension [10], are well documented in meta-analyses. However, the direct relationship between CVD and cataracts remains incompletely understood from past epidemiological studies. Bridging this knowledge gap requires a systematic review to synthesize evidence on whether individuals with established CVD face an increased risk or odds of developing cataracts or requiring cataract surgery. This review will attempt to pool all published evidence on observational studies and determine the associations between cataracts and CVD. Thus, clinicians will be able to leverage the pooled evidence to develop integrated care models that simultaneously address ocular and cardiovascular health. For example, preventive ophthalmology strategies will incorporate cardiovascular risk screening in patients with cataracts to promote early detection and management of CVD. To our knowledge, there is currently no published systematic review that examines our specific research question: Do adults with established CVD have a higher risk or likelihood of developing cataracts or of requiring cataract surgery compared to those without CVD?

The primary objective of this study is to determine the risk or likelihood of developing cataracts or requiring cataract surgery among adults with CVD. This review will include all relevant published studies, including cross-sectional and cohort studies irrespective of publication year. By synthesizing evidence from diverse study designs, we aim to identify the impacts of CVD on cataract development.

## Methods

### Overview

We will adhere to the guidelines outlined in the PRISMA (Preferred Reporting Items for Systematic Reviews and Meta-Analyses) checklist and its extension for protocols (Preferred Reporting Items for Systematic Reviews and Meta-Analyses Protocols [PRISMA-P]) (Checklist 1) [11]. This review has been registered with PROSPERO (CRD42025629907) on January 16, 2025, and the Ministry of Health Malaysia’s National Medical Research Register under the registration number NMRR ID-24-03414-99G on October 22, 2024. No protocol amendments were made.

### Formulation of the Research Question

The research question was developed using the population, exposure, comparator, and outcome framework [12], with adults (aged ≥18 years) as the study population. The exposure of interest was established CVD, while individuals without established CVD served as the comparison group. The outcome was the risk or odds of cataract onset or surgery.

### Data Source and Search Strategy

A systematic electronic literature search will be conducted across 4 major databases: PubMed, Scopus, Web of Science, and Embase as they cover extensively indexed, peer-reviewed articles from diverse health science sources. Gray literature, namely, Google Scholar, will not be explored due to potential inclusion of non–peer-reviewed content. The search will cover all published studies from database inception to December 31, 2024, using search terms classified into 2 main concepts: CVD and cataracts. The keywords (Table 1) will be derived from the main components of the research question. A combination of MeSH terms and free-text terms will be used, targeting the title and abstract fields. Additionally, cross-referencing involving screening the reference lists of relevant articles for additional sources will also be conducted.

**Table 1.** Keywords used in the search process.

Databases	Keywords
PubMed	((cardiovascular diseases) OR (vascular diseases) OR (heart diseases) OR (acute coronary syndrome) OR (coronary disease) OR (myocardial ischemia) OR (myocardial infarction) OR (coronary artery disease) OR (stroke) OR (cerebrovascular disorders) OR (ischemic stroke) OR (hemorrhagic stroke) OR (transient ischemic attack) OR (heart failure) OR (heart decompensation) OR (congestive heart failure) OR (ventricular dysfunction) OR (embolism and thrombosis) OR (venous thrombosis) OR (pulmonary embolism) OR (peripheral arterial disease) OR (peripheral vascular diseases)) AND ((cataract) OR (cataract surgery) OR (phacoemulsification))
Scopus	(“cardiovascular diseases” OR “vascular diseases” OR “heart diseases” OR “acute coronary syndrome” OR “coronary disease” OR “myocardial ischemia” OR “myocardial infarction” OR “coronary artery disease” OR stroke OR “cerebrovascular disorders” OR “ischemic stroke” OR “hemorrhagic stroke” OR “transient ischemic attack” OR “heart failure” OR “heart decompensation” OR “congestive heart failure” OR “ventricular dysfunction” OR “embolism and thrombosis” OR “venous thrombosis” OR “pulmonary embolism” OR “peripheral arterial disease” OR “peripheral vascular diseases”) AND (cataract OR (cataract surgery) OR (phacoemulsification))
Web of Science	(TS=(“cardiovascular disease”) OR TS=(“vascular diseases”) OR TS=(“heart diseases”) OR TS=(“acute coronary syndrome”) OR TS=(“coronary disease”) OR TS=(“myocardial ischemia”) OR TS=(“myocardial infarction”) OR TS=(“coronary artery disease”) OR TS=(stroke) OR TS=(“cerebrovascular disorders”) OR TS=(“ischaemic stroke”) OR TS=(“hemorrhagic stroke”) OR TS=(“transient ischemic attack”) OR TS=(“heart failure”) OR TS=(“heart decompensation”) OR TS=(“congestive heart failure”) OR TS=(“ventricular dysfunction”) OR TS=(embolism) OR TS=(thrombosis) OR TS=(“pulmonary embolism”) OR TS=(“venous thrombosis”) OR TS=(“peripheral arterial disease”) OR TS=(“peripheral vascular diseases”)) AND (TS=(cataract) OR TS=(“incident cataract”) OR TS=(“cataract surgery”) OR TS=(phacoemulsification))
Embase	(“cardiovascular disease”/exp OR “cardiovascular disease” OR “vascular diseases”/exp OR “vascular diseases” OR (vascular AND (“diseases”/exp OR diseases)) OR “heart diseases”/exp OR “heart diseases” OR ((“heart”/exp OR heart) AND (“diseases”/exp OR diseases)) OR “acute coronary syndrome”/exp OR “acute coronary syndrome” OR (acute AND coronary AND (“syndrome”/exp OR syndrome)) OR “coronary disease”/exp OR “coronary disease” OR (coronary AND (“disease”/exp OR disease)) OR “myocardial ischemia”/exp OR “myocardial ischemia” OR (myocardial AND (“ischemia”/exp OR ischemia)) OR “myocardial infarction”/exp OR “myocardial infarction” OR (myocardial AND (“infarction”/exp OR infarction)) OR “coronary artery disease”/exp OR “coronary artery disease” OR (coronary AND (“artery”/exp OR artery) AND (“disease”/exp OR disease)) OR “stroke”/exp OR stroke OR “cerebrovascular disorders”/exp OR “cerebrovascular disorders” OR (cerebrovascular AND (“disorders”/exp OR disorders)) OR “ischemic stroke”/exp OR “ischemic stroke” OR (ischemic AND (“stroke”/exp OR stroke)) OR “hemorrhagic stroke”/exp OR “hemorrhagic stroke” OR (hemorrhagic AND (“stroke”/exp OR stroke)) OR “transient ischemic attack”/exp OR “transient ischemic attack” OR (transient AND ischemic AND attack) OR “heart failure”/exp OR “heart failure” OR ((“heart”/exp OR heart) AND (“failure”/exp OR failure)) OR “heart decompensation”/exp OR “heart decompensation” OR ((“heart”/exp OR heart) AND decompensation) OR “congestive heart failure”/exp OR “congestive heart failure” OR (congestive AND (“heart”/exp OR heart) AND (“failure”/exp OR failure)) OR “ventricular dysfunction”/exp OR “ventricular dysfunction” OR (ventricular AND dysfunction) OR ((“embolism”/exp OR embolism) AND (“thrombosis”/exp OR thrombosis)) OR “venous thrombosis”/exp OR “venous thrombosis” OR (venous AND (“thrombosis”/exp OR thrombosis)) OR “pulmonary embolism”/exp OR “pulmonary embolism” OR (pulmonary AND (“embolism”/exp OR embolism)) OR “peripheral arterial disease”/exp OR “peripheral arterial disease” OR (peripheral AND arterial AND (“disease”/exp OR disease)) OR “peripheral vascular diseases”/exp OR “peripheral vascular diseases” OR (peripheral AND vascular AND (“diseases”/exp OR diseases))) AND (“cataract”/exp OR cataract OR “cataract surgery” OR phacoemulsification)

Cross-sectional studies will be analyzed separately from cohort studies because the former assess associations at a single point in time and cannot establish temporality or determine the risk of developing cataracts. Cohort studies will be used to assess the risk of incident cataracts following exposure to CVD. Furthermore, cataract diagnosis and cataract surgery will be considered distinct outcomes as cataract surgery represents a subsequent clinical intervention rather than the diagnosis of cataracts themselves.

### Eligibility Criteria

The eligibility criteria are outlined in Textbox 1.

Textbox 1. **Inclusion and exclusion criteria.**

**Inclusion criteria**

- Published materials from inception to December 31, 2024
- English-language publications
- Original research articles
- Studies involving adult participants (aged ≥18 y)
- Studies assessing statistical relationships between baseline cardiovascular disease (operational definition includes acute coronary syndrome, stroke, deep vein thrombosis and pulmonary embolism, congestive cardiac failure, or peripheral arterial disease) [13] and incident cataracts or cataract surgery

**Exclusion criteria**

- Conference proceedings, commentaries, reports, reviews, dissertations, editorials, conference abstracts, or non–peer-reviewed articles
- Studies involving non–age-related cataracts, such as ocular damage causing traumatic cataracts or systemic diseases causing uveitic cataracts

### Study Selection

Two authors will independently screen the titles and abstracts based on the eligibility criteria. Another 2 authors will then retrieve the full texts and enter the data into Microsoft Excel. Any discrepancies will be discussed between the reviewers, and unresolved issues will be addressed through consultation with a third researcher. Articles deemed irrelevant will be excluded. The full texts of the remaining articles will be retrieved for detailed assessment. Authors of the included studies may be contacted for additional details if necessary.

### Quality Appraisal

All included studies will be assessed using the Newcastle-Ottawa Scale (NOS), a tool designed to evaluate the quality of nonrandomized studies [13]. The original NOS for cohort studies and case-control studies will be applied for longitudinal and case-control designs, respectively. For cross-sectional studies, the modified NOS will be used [14]. Each form uses a star scoring system to assess key aspects of the study. The scoring system evaluates studies across 3 main domains: selection, comparability, and exposure (for case-control studies) or outcome (for cohort studies).

The assessment will be conducted based on the following domains. For cohort studies, the domains include selection (maximum of 4 stars), comparability (maximum of 2 stars), and outcome assessment (maximum of 3 stars). For case-control studies, the domains include selection (maximum of 4 stars), comparability (maximum of 2 stars), and exposure assessment (maximum of 3 stars). For cross-sectional studies, the domains include selection (maximum of 5 stars), comparability (maximum of 2 stars), and outcome or exposure assessment (maximum of 3 stars).

Each included study is scored based on these predefined criteria, with higher scores indicating better methodological quality. Studies with an NOS score of less than 3 are classified as being of poor quality, those with scores between 3 and 6 are considered of moderate quality, and studies with scores of 7 or higher are classified as being of high quality.

### Data Extraction and Synthesis

Data will be extracted into a standardized Microsoft Excel form by 2 authors and summarized in an evidence table. Extracted information will include authors; publication year; country; study design; study objective; age of recruited participants; sample size; types of CVD; measured outcomes (cataract subtype); and results of statistical analysis, including adjusted potential confounders (if any).

Discrepancies in data extraction will be resolved through discussion between reviewers or consultation with a third researcher when consensus cannot be reached. Data synthesis will use content analysis to group data into fewer content-related categories.

### Meta-Analysis

A random-effects meta-analysis will be performed via the Cochrane software Review Manager for studies with homogeneous populations, interventions, and outcomes [15]. Statistical data will be presented as relative risks or odds ratios with 95% CIs depending on the study design. Heterogeneity will be assessed using forest plots and the *I*^2^ statistic. An *I*^2^ value of 75% or higher indicates high heterogeneity, prompting further investigation into sources of variability [16]. Publication bias will be assessed via visually inspecting funnel plots, complemented by the Egger test or Begg test where applicable.

If the included studies exhibit different characteristics, such as variations in cataract subtypes (namely, posterior subcapsular, cortical, and nuclear), subgroup analyses will be performed to account for these differences. This statistical approach allows for a more detailed understanding of how specific types of cataracts may be associated with underlying CVD. By analyzing these subgroups, this review aims to identify differential effects that may otherwise be obscured in an overall pooled analysis.

## Results

This study did not receive any external funding. The literature search, study screening, and data extraction will be conducted in 2026, and data analysis will be performed accordingly. The findings are anticipated to be published in 2027.

## Discussion

The review and meta-analysis will systematically summarize the evidence on the association between CVD and cataracts. It is anticipated that individuals with CVD may have an increased risk of cataracts compared to those without CVD. However, the magnitude and consistency of this association may vary according to differences in study populations, definitions of CVD and its subtypes, cataract classification, and methodological characteristics. Therefore, this review will not only quantify the overall association but also explore potential sources of variability across studies.

There may be inconsistent associations observed across different eligible studies, which may be attributed to variations in the methodologies used to assess CVD. Notably, an important source of heterogeneity is likely to arise from differences in the operational definitions of CVD and its subtypes. For example, some studies may rely on self-reported histories of CVD, which, while practical for large-scale surveys, may introduce misclassification or variability in outcome ascertainment [17]. Similarly, differences in cataract classification may affect pooled estimates of the association. While some studies may use standardized grading systems such as the Lens Opacities Classification System III (LOCS III), others may rely on self-reported cataract status, medical records, or less standardized clinical assessments. The LOCS III provides standardized grading of nuclear, cortical, and posterior subcapsular lens opacities and has demonstrated good interobserver agreement [18]. However, in real-world clinical practice, where time and clinic workload constraints may limit the feasibility of systematic grading, cataract assessment may not always strictly adhere to LOCS III criteria. Such differences in ascertainment may lead to misclassification bias, particularly when distinguishing between specific cataract subtypes, and, consequently, contribute to variability in the magnitude and direction of the observed associations.

Additionally, considerable heterogeneity may arise from differences at both the clinical and methodological levels [19]. Clinical heterogeneity may reflect differences in participant characteristics, the CVD outcomes investigated, and the ascertainment methods used for exposures and outcomes. Methodological heterogeneity may additionally arise from differences in study design and analytical methods, including prospective vs retrospective designs, duration of follow-up, and the extent of adjustment for potential confounding factors [19]. Potential sources of heterogeneity will be explored through subgroup analyses [20-22]. This approach may help identify factors contributing to heterogeneity and determine whether the magnitude of the association differs across clinically or methodologically distinct groups [19].

Differences in the prevalence and distribution of medical comorbidities across study populations may further contribute to heterogeneity in the observed associations. Although many studies may adjust for established confounders such as diabetes mellitus and hypertension, differences in the extent and quality of confounder adjustment may result in residual confounding. This may be particularly relevant in older populations, in whom CVD and cataracts share several age-related and metabolic risk factors. The potential influence of age may reflect shared biological processes, including cumulative oxidative stress or vascular dysfunction, which are implicated in both CVD and age-related cataract formation [23]. Geographical differences are also expected to play a significant role in these inconsistencies. For example, UV radiation exposure is a well-recognized environmental risk factor for cortical cataracts and varies substantially according to geographical location, occupation, and lifestyle [24,25]. Studies conducted in high–sun exposure regions, such as the landmark Blue Mountains Eye Study in Australia [26], have emphasized the role of occupational sun exposure in cataract risk. In contrast, this environmental factor may be less relevant in regions with cooler climates and lower levels of UV radiation.

To enhance the methodological rigor and transparency of the review, this protocol has been developed in accordance with relevant systematic review reporting and methodological guidance, including the PRISMA-P framework. A comprehensive search strategy was developed using predefined eligibility criteria and more than 70 keywords to search 4 databases: PubMed, Scopus, Web of Science, and Embase. To ensure comprehensive and systematic identification of relevant studies, the retrieved records will be deduplicated and screened according to the predefined eligibility criteria. In addition, a formal risk-of-bias assessment will be conducted using the original NOS for cohort and case-control studies and the modified NOS for cross-sectional studies, both of which are widely used instruments for assessing the risk of bias in observational studies [27]. The use of these structured assessment tools provides a practical and standardized approach to evaluating the methodological quality of the included studies while remaining feasible within a review process involving at least 2 reviewers [28].

Despite these methodological strengths, several potential limitations should be acknowledged. First, restricting inclusion to peer-reviewed studies may result in the omission of unpublished or non–peer-reviewed evidence [29]. Consequently, publication bias may remain a concern, particularly if studies reporting null or nonsignificant associations are less likely to be published [30]. Second, restricting inclusion to English-language publications may introduce language bias and result in the omission of relevant evidence published in other languages. Third, the inclusion of cross-sectional studies may limit causal inference because exposure and outcome are assessed at approximately the same point in time, thereby limiting the ability to establish temporality. Thus, pooled estimates should be interpreted as measures of association rather than as evidence of a causal relationship.

If a consistent association between CVD and cataracts is identified, the findings may have implications for clinical practice [31], including greater consideration of cardiovascular risk assessment among patients presenting with cataracts. However, the evidence should be interpreted cautiously before recommending routine cardiovascular screening solely based on cataracts. Nevertheless, the age-related nature of both CVD and cataracts may introduce potential confounding as the observed association between these conditions may be partly influenced by the effects of aging. From a public health perspective, these findings can guide preventive strategies aimed at reducing the burden of both noncommunicable diseases in aging populations. Clinically, evidence of a consistent association may support a more integrated multidisciplinary approach involving ophthalmologists, primary care physicians, and cardiologists, particularly for patients with multiple shared cardiovascular and ocular risk factors.

In conclusion, this systematic review and meta-analysis will synthesize the available evidence on the association between CVD and cataracts. The review is expected to provide a clearer understanding of this relationship by quantifying the associations, evaluating the consistency of the findings, and identifying potential sources of heterogeneity. These findings may help inform priorities for future epidemiological and clinical research.

## Supplementary material

10.2196/96552 Checklist 1 PRISMA-P checklist.

## Abbreviations

CVD: cardiovascular disease
LOCS III: Lens Opacities Classification System III
NOS: Newcastle-Ottawa Scale
PRISMA: Preferred Reporting Items for Systematic Reviews and Meta-Analyses
PRISMA-P: Preferred Reporting Items for Systematic Reviews and Meta-Analyses Protocols
